# High-dimensional multiplexing through vortex electromagnetic wave manipulation by space-time-coding metasurfaces

**DOI:** 10.1038/s41377-026-02232-6

**Published:** 2026-03-09

**Authors:** Chenfeng Yang, Si Ran Wang, Jia Chen Du, Man Ting Wang, Zheng Xing Wang, Ka Fai Chan, Dong-Ze Zheng, Geng-Bo Wu

**Affiliations:** 1 State Key Laboratory of Terahertz and Millimeter Waves, City University of Hong Kong, Hong Kong, 999077 China; 2 Department of Electrical Engineering, City University of Hong Kong, Hong Kong, 999077 China; 3 Department of Electrical and Computer Engineering, University of Alberta, Edmonton, AB T6G 2R3 Canada; 4 School of Electronics Science and Engineering, University of Electronics Science and Technology of China, Chengdu, 611731 China

**Keywords:** Metamaterials, Electronics, photonics and device physics

## Abstract

Orbital angular momentum (OAM) is a fundamental property of light, with widespread applications across various fields, from quantum mechanics to advanced imaging and telecommunications. The inherent orthogonality of OAM beams allows information multiplexing in unique, high-dimensional states. However, conventional OAM-based systems encounter long-standing integration and scalability challenges due to the heavy reliance on complex optical components and redundant radio frequency chains for multi-channel transmission. Here, we propose a dual-polarized asynchronous space-time-coding metasurface (DASM) for producing coaxial OAM beams in multiple physical domains through a single aperture. By synergistically combining OAM, polarization, and frequency division multiplexing, DASM constructs a high-dimensional communication framework, dramatically increasing the number of supported channels. Notably, DASM further streamlines the framework by directly modulating the information carried by OAM beams, eliminating the need for complicated external modulators. The high-dimensional multiplexing framework offers a simplified, versatile, and efficient solution for substantial development in wireless communications capacity and scalability.

## Abstract (web summary)

A programmable metasurface enables direct information modulation and high-dimensional communications by multiplexing OAM, polarization, and frequency.

## Graphical abstract

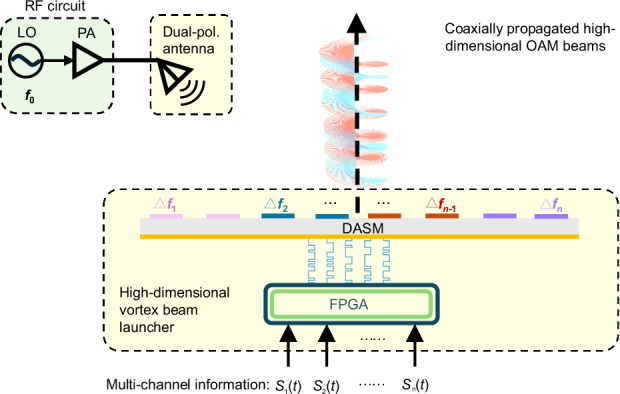

## Introduction

Multiplexing technologies serve as the linchpin of modern wireless communications, enabling the efficient utilization of limited resources to meet the exponentially growing demand for higher data rates. Conventional multiplexing techniques, such as frequency, time, code, and polarization division multiplexing, have been widely employed to maximize wireless communication capacity. Recently, orbital angular momentum (OAM) mode division multiplexing has emerged as a promising approach, introducing a new physical dimension for wireless communications. As one of the fundamental properties of electromagnetic (EM) waves, OAM is characterized by the mode index *ℓ* that represents the degree of beam vorticity and phase singularity. Theoretically, different OAM modes are mutually orthogonal, and the information they encode does not interfere with each other. In the line-of-sight wireless communications, the transmitted vortex beams can conceptually carry infinite OAM modes, thereby supporting multiple independent data streams with minimal crosstalk^1–7^.

Significant challenges exist in practical OAM-based wireless communication systems, i.e., difficulties in generating multiplexed OAM beams and cumbersome system architecture. Common vortex beam generation techniques in the open literature include spiral phase plates^8^, cylindrical lenses^9^, uniform circular antenna arrays^10^, annular traveling-wave antenna^11^, or spoof surface plasmons^12^, etc. However, these techniques inherently support only fixed OAM states due to their passive structures. The number of available communication channels is constrained by the predefined OAM modes, limiting the scalability and adaptability required for future ultra-fast and high-capacity wireless communication systems.

Moreover, OAM technology holds an even greater promise than its current applications. Inspired by the concept of high-dimensional multiplexing^13–16^, OAM can be integrated with additional multiplexing techniques for even more communication channels. Other physical properties of EM waves can be utilized, such as frequency^17,18^, and polarization^19–21^ to tailor multi-mode OAM beams in multiple dimensions^22^. High-dimensional multiplexing is implemented by encoding information onto these OAM beams, thereby dramatically enhancing communications capacity and spectral efficiency. However, achieving this ambitious goal requires simultaneous and efficient control of multiple intrinsic EM wave properties, including amplitude, phase, polarization, momentum, and frequency, which remains a great challenge. Conventional high-dimensional systems rely on multi-port antennas or dedicated hardware for each dimension of multiplexing^23–25^, leading to bulky communications devices, increased system complexity, and high power consumption.

Owing to the rapid development of metasurfaces, new possibilities of high-dimensional multiplexing have emerged. As a thin 2D structure composed of subwavelength meta-atoms, metasurfaces offer a low-cost, lightweight, and efficient solution for manipulating multiple EM wave properties^26–32^. By incorporating time modulation into conventional programmable metasurface design, space-time-coding metasurfaces (STCMs) are distinguished for dynamically controlling EM wave properties in both spatial and time domains. The customized scattering characteristics in each period allow STCMs to generate several harmonics, where the phase and amplitude of each harmonic can be continuously controlled^33–40^. Although the previous study^37^ demonstrates a STCM antenna capable of manipulating all fundamental properties of EM waves, its one‑dimensional linear‑array configuration and asymmetric meta‑atom design lead to non‑uniform azimuthal sampling, making high‑purity and multi-mode OAM beam generation challenging. Consequently, the use of STCMs for multi-mode OAM beam generation, direct information modulation, and high‑dimensional multiplexing remains elusive.

Here, we propose a novel dual-polarized asynchronous STCM (DASM) capable of achieving high-dimensional OAM communications through several multiplexing degrees. Based on external control sequences, DASM facilitates the generation of multiple OAM beams in real-time and directly encodes phase and amplitude information onto them. Furthermore, DASMs can generate OAM beams with distinct linear polarizations (LPs) through specifically engineered meta-atom architecture. One inherent challenge of STCMs is achieving independent frequency manipulation, as different harmonics often exhibit strong entanglement. The asynchronous control mechanism is utilized to achieve frequency division multiplexing. The universal EM control capability makes the proposed DASM an attractive solution for implementing high-dimensional multiplexing, where precise, independent, and flexible control of EM wave properties is essential.

Our experimental results demonstrate that the proposed DASM enables complex wavefront manipulation to dynamically generate vortex beams with numerous OAM modes at different LP states and frequencies. We further show that our DASM supports multiple channels of Quadrature Phase Shift Keying (QPSK) communications by multiplexing across OAM, polarization, and frequency dimensions. These findings highlight the potential of the proposed DASM integrated with multiple multiplexing techniques for high-dimensional communication frameworks.

## Results

### High-dimensional vortex beam generation

**Fig. 1:**
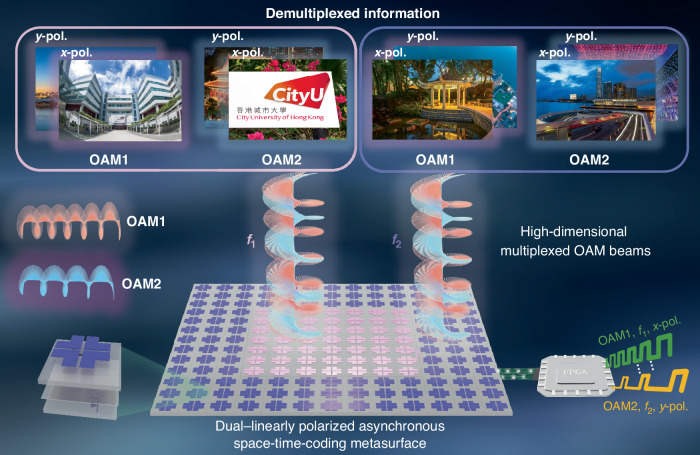
Concept of the high-dimensional multiplexing based on the dual-polarized asynchronous space-time-coding metasurface. The space-time-coding sequences implemented in the FPGA are used to synthesize multi-mode OAM beams at multiple polarizations and frequencies. The metasurface aperture is partitioned into two distinct regions, marked by different colors, each responsible for OAM beam generation at separate carrier frequencies. The dual LP meta-atom is designed for wave manipulation at different linear polarizations

As illustrated in Fig. 1, the proposed high-dimensional communication framework employs the DASM to simultaneously generate and manipulate multi-mode OAM beams across diverse polarizations and frequencies. The metasurface comprises a 12 × 12 array of reflective meta-atoms (left corner of Fig. 1). Two positive-intrinsic-negative (PIN) diodes are mounted in the slots of the top metal pattern, allowing independent EM wave control along both *x-* and *y-*polarizations. For each polarization, the reflection phase state can be switched between “0” and “π” with consistent amplitude by applying an external voltage excitation. To achieve independent beam manipulation at multiple frequencies, we partitioned the metasurface into two distinct partitions that generate separate groups of frequency harmonics. These partitions operate as independent EM wave modulators, supporting the concurrent transmission of vortex beams at different frequencies ^41^.

**Fig. 2:**
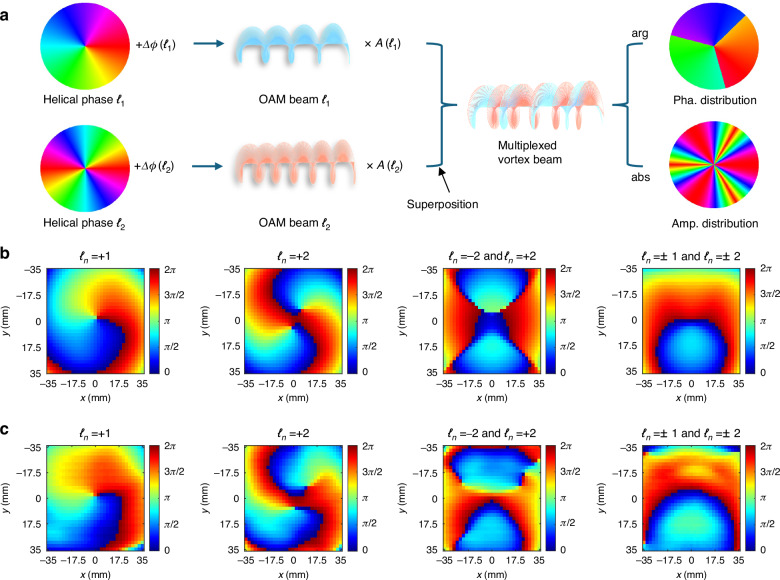
Multi-mode OAM generation and multiplexing. **a** Schematic illustration of obtaining the required phase and amplitude distributions for generating an information-carrying vortex beam with multiple OAM modes. **b** Calculated near-field phase distributions for four typical vortex beams. **c** Measured near-field phase distributions for four typical vortex beams

Figure 2a unravels the underlying physics of OAM mode division multiplexing. We begin by generating a single-mode OAM beam with broadside radiation at a specific frequency and polarization. The *ℓ*_*n*_-th mode OAM beam involves a helical phase distribution, denoted as *ℓ*_*n*_∙arctan(*y*/*x*), where (*x*, *y*) is the Cartesian coordinate. The corresponding surface E-field can be expressed as follows^42^:

1 $$E\left({\ell }_{n}\right)={e}^{i\cdot [{\ell }_{n}\cdot arctan\left(\frac{y}{x}\right)+\Delta \phi \left({\ell }_{n}\right)]}$$

where *Δϕ*(*ℓ*_*n*_) represents the phase information encoded onto the *ℓ*_*n*_-th OAM mode. By applying the principles of multivorticity metasurfaces^43,44^, the vortex beam carrying multiple OAM modes can be generated by superimposing their respective surface E-fields. Moreover, the intensity proportions of OAM in different modes can be freely adjusted, allowing the amplitude information *A*(*ℓ*_*n*_) to be encoded onto the vortex beam. The resultant phase and amplitude distributions for the metasurface are then retrieved from the superpositioned E-field, as follows:

2 $$\left\{\begin{array}{l}{Pha}\,\left(x,y\right)=arg\left[\mathop{\sum}\limits_{n{=}{1}}^{m}\sqrt{{A}({\ell}_{n})}{\times}E{(}{\ell }_{n}{)}\right]\\ {Amp}\,(x,y)=abs\left[\mathop{\sum}\limits_{n{=}{1}}^{m}\sqrt{{A}({\ell}_{n})}{\times }E{(}{\ell}_{n}{)}\right]\end{array}\right.$$

Importantly, the encoded amplitude and phase information of the vortex beam remains preserved after E-field superimposition. In practical wireless communications, the conveyed information can be accurately decoded independently across multiple channels through OAM demultiplexing.

As is evident from Eq. 2, the generation of the vortex beam carrying multiple OAM modes requires sufficient and independent engineering of the surface’s phase and amplitude distributions^45^. However, conventional programmable metasurfaces loaded with PIN/varactor diodes face challenges in achieving the necessary continuous and independent control of phase and amplitude. Aiming at solving this limitation, STCMs are introduced for quasi-continuous and independent manipulation of both phase and amplitude distributions. In this paradigm, the reflectivity of the meta-atoms is periodically modulated according to a pre-defined time-coding sequence. Assuming the period of the sequence is *T*_0_ = 1/*Δf*, *Δt* is the time delay of phase state “π” in one period, and *M* is the duty ratio for phase state “π”. The complex amplitude of each meta-atom can be derived from the Fourier series of the time-modulated reflectivity^46^:

3 $$\left\{\begin{array}{l}{\varphi }_{k}=-k{\omega }_{0}\varDelta t-\frac{\pi }{2}\left[1-{\left(-1\right)}^{\left\lfloor \left|k\right|\cdot M\right\rfloor }\right],\,0\le \varDelta t < \frac{{T}_{0}}{\left|k\right|}\\ {A}_{k}=2{M|Sa}\left(k\pi M\right)|,\,0\le M\le \frac{1}{2\left|k\right|}\end{array}\right.$$

where *Sa*(·) indicates the *sinc* function, and ⌊·⌋ denotes the rounding down operation. It can be observed that the amplitude and phase of any specific harmonic could be manipulated independently and continuously by adjusting duty ratios *M* and time delays *Δt* of the input sequences, respectively (Details in Supplementary Note S1). Meanwhile, the meta-atoms at different positions are independently controlled by distinct time-coding sequences, thus constituting spatial-temporal modulation (STM). Afterwards, arbitrary amplitude and phase distributions across the metasurface aperture can be synthesized, facilitating the efficient generation of complex beam shapes.

Without loss of generality, we take the +1^st^ harmonic as an example in this research. A 2 × 2 magnetoelectric dipole antenna array is employed as the feed source to radiate the fundamental 26.8 GHz monochromatic wave. The configuration and performance of the source are shown in Supplementary Note S2. By applying a STM frequency Δ*f* of 100 kHz, the +1st harmonic is shifted to 26.8 GHz + 100 kHz. To validate DASM’s multi-mode OAM beam generation performance, the near-field measurement experimental setup is configured in a microwave anechoic chamber, and the details are presented in Supplementary Note S3. Specifically, OAM modes with indices *ℓ*_*n*_ = ±1, ±2 are generated either independently or simultaneously through carefully designed STM sequences. The input sequences corresponding to the 144 meta-atoms are organized into a matrix, as illustrated in Supplementary Fig. S11. The near-field E-field distribution is measured at a transverse plane located 81 mm away from the metasurface aperture, which has a square area with a side length of 70 mm. The measurement plane is discretized with a step size of 2.33 mm for adequate spatial resolution.

Figure 2b, c shows the experimental results for four typical vortex beams, including single OAM mode (*ℓ*_*n*_ = +1 and *ℓ*_*n*_ = +2) as well as superimposed OAM modes (*ℓ*_*n*_ = ±2 as well as *ℓ*_*n*_ = ±1 and ±2). It can be observed that the phase distribution at the transverse plane undergoes a one-cycle rotation for *ℓ*_*n*_ = +1, while a two-cycle rotation is observed in *ℓ*_*n*_ = +2. These distinct rotational phase patterns ensure that the information carried by OAM mode *ℓ*_*n*_ can only be decoded by a corresponding receiving OAM antenna with an inverse phase mask. The doughnut shape amplitude distributions can be observed in Supplementary Fig. S5. The slight E-field distortion of the measured wavefront can be attributed to the feed blockage of the reflective-type metasurface and imperfections of the metasurface fabrication. In general, the measured phase distributions are in reasonable agreement with the calculated results. All these near-field experimental results demonstrate DASM’s ability to generate and dynamically manipulate multi-mode OAM beams. Moreover, the proposed DASM can utilize its dual-LP structure and asynchronous modulation strategy to shape the EM wavefronts across multiple polarizations and frequencies, thereby constructing a high-dimensional vortex beam launcher.

### OAM-based mode division multiplexing transmitter

**Fig. 3:**
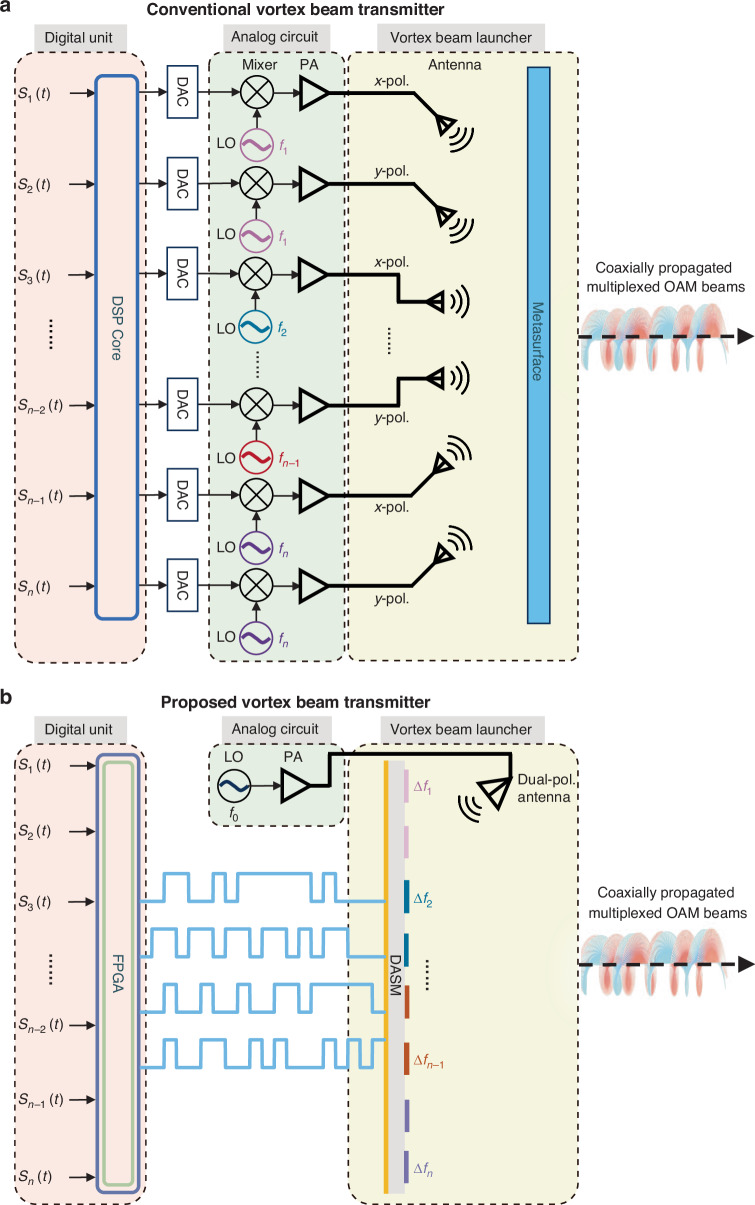
Architectures of OAM multiplexing transmitters. **a** Block diagram of a conventional vortex beam transmitter. **b** Schematic diagram of the proposed high-dimensional multiplexing transmitter using the proposed DASM with simplified and compact architecture

We design a DASM-based transmitter to demonstrate the advantages of vortex beam manipulation across OAM modes, polarizations, and frequencies. Figure 3 compares two multi-OAM-channel transmitter architectures for wireless communications. The first one, depicted in Fig. 3a, is the conventional OAM transmitter architecture that employs passive metasurfaces to achieve OAM mode division multiplexing^47–51^. However, the functionality of these metasurfaces is restricted to OAM beam generation, and the transmitter still relies on multiple complex and power‑hungry RF chains to upconvert the baseband signals to RF frequencies. To be specific, each OAM channel must be supported by a dedicated RF chain, including digital‑to‑analog converters (DACs), mixers, and local oscillators (LOs). This inevitably increases hardware cost and system complexity. Moreover, multiple RF sources should be physically separated into different positions, further enlarging the system footprint. By contrast, our proposed DASM-based transmitter (Fig. 3b) not only generates multiplexed OAM beams under a simple monochromatic continuous-wave excitation but also directly modulates the information (i.e., baseband message) into the OAM waves. Our DASM can directly generate the required modulated EM waveforms driven by the digital control signals from an FPGA, eliminating the need for extra high-speed DACs and mixers for communication channels. The reduced number of RF chains and components significantly decreases the hardware overhead.

In terms of signal modulation, DASM is able to manipulate the phase and amplitude information of each constituent OAM mode independently. The key to achieving direct information modulation across different channels is to manipulate the aperture’s amplitude and phase distribution in real-time. From this viewpoint, an alternative candidate for the simplified OAM multiplexing system is phased array. A traditional millimeter-wave phased array relies on phase shifters and amplifiers per element for complex E-field control. These active RF components typically consume power on the order of several tens to a few hundred mW per element^52–54^. In our DASM system, each PIN diode (MACOM MADP 000907 14020x) consumes less than 10 mW of power. Therefore, our proposed DASM is expected to offer a significantly simplified hardware architecture with enhanced communications capacity and low power consumption for OAM multiplexing transmitters.

On the receiving side, the information from the transmitted high-dimensional OAM beam can be demultiplexed one at a time using an OAM lens antenna receiver. To extract the information of a specific OAM component *ℓ*_*n*_, an OAM lens antenna with inverse azimuthal phase profile is employed to eliminate the azimuthal helical phase distribution. This compensation effectively unwinds the target OAM component, transforming it into the fundamental mode (*ℓ*_*n*_ = 0). Then, the energy of the target OAM mode is efficiently focused at the focal point of the lens, where the carried information can be detected by a dual-LP receiving horn antenna. For the other OAM modes that do not match the configuration of the inverse OAM lens antenna, the helical phase distribution cannot be completely compensated. The remaining helical wavefront causes a radiation null in the propagation direction. As a result, the receiving antenna cannot receive the signal under mismatched demultiplexing conditions. Theoretical analysis and design details for the OAM demultiplexing lens are provided in Supplementary Note S5.

**Fig. 4:**
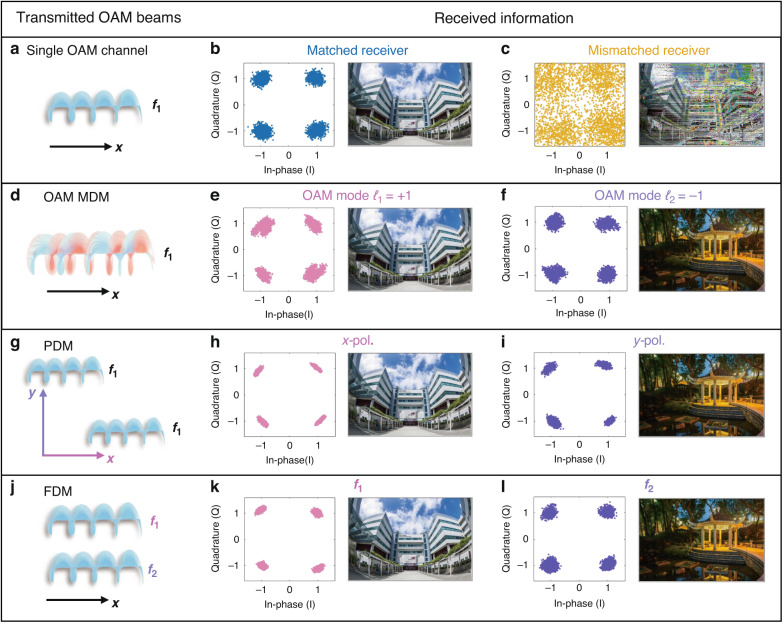
Constellation diagrams and the corresponding recovered images in OAM-based wireless communications. **a**–**c** Single OAM channel with matched and mismatched receivers. **d**–**f** OAM mode division multiplexing (MDM) with receiver modes *ℓ*_1_ = +1 and *ℓ*_2_ = −1. **g**–**i** Polarization division multiplexing (PDM) with *x*-polarization and *y*-polarization channels. **j**–**l** Frequency division multiplexing (FDM) with frequencies 26.8001 GHz and 26.80025 GHz

Figure 4a–c depicts the experimental results of successful and unsuccessful OAM communications at a carrier frequency of 26.8001 GHz. On the transmitting side, an OAM beam of mode *ℓ*_*n*_ = +1 is generated carrying the information of a picture encoded in QPSK format with a transmission symbol rate of 50 kbps. The symbol rate for each QPSK channel can be increased by optimizing the FPGA control circuits, as discussed in detail in Supplementary Note S8. In Fig. 4b, the receiving horn antenna is equipped with a corresponding demultiplexing lens with inverse azimuthal phase profile. Under this matched situation, the decoded QPSK constellation points are distinguishable, and the picture is successfully recovered without noise points. Conversely, Fig. 4c shows the scenario with a mismatched demultiplexing lens that has the same azimuthal phase profile. In this scenario, the energy coupled to the receiving antenna is minimal. As a result, the quality of the decoded QPSK constellations is significantly deteriorated, leading to a distorted received image.

Afterwards, we validate the feasibility of OAM mode division multiplexing under single-frequency and single-polarization conditions. As illustrated in Fig. 4d, the transmitted waves comprise a vortex beam carrying two distinct OAM modes, specifically *ℓ*_1_ = + 1 and *ℓ*_2_ = -1, each serving as an independent communication channel to transmit the information of a unique image. In Fig. 4e, the information is retrieved using a demultiplexing lens *ℓ*_1_ = + 1. The demultiplexing lens cancels the vortex of OAM mode *ℓ*_1_ = + 1 while enhancing the vortex of OAM mode *ℓ*_2_ = -1. As a result, only the information associated with OAM mode *ℓ*_1_ is decoded, while the contribution of OAM mode *ℓ*_2_ is suppressed. The decoded constellation diagram reveals that the data points are stably distributed at the four corners, forming four clusters with well-defined boundaries. Similarly, Fig. 4f displays the information obtained by replacing the demultiplexing lens with one configured for *ℓ*_2_ = -1. In this case, the receiver successfully decodes the information associated with OAM mode *ℓ*_2_, producing a clear and distinguishable constellation diagram. Moreover, two different images are successfully recovered through the two channels in Fig. 4e, f, demonstrating the low crosstalk between two channels and the effectiveness of OAM-based communications.

To further demonstrate that OAM mode division multiplexing can be integrated with other multiplexing technologies, we performed a series of experimental evaluations by loading the OAM beam with mode *ℓ*_*n*_ = +1 into traditional polarization and frequency division multiplexing systems. As a preliminary step, we validate the compatibility of integrating the OAM beam into a dual-LP communication system, as shown in Fig. 4g. In this case, a dual-LP source was employed to illuminate DASM, generating two OAM beams with the same mode *ℓ*_*n*_ = +1 at a carrier frequency of 26.8001 GHz. Each beam is independently modulated with distinct image information at orthogonal polarization states, respectively. For the receiver side, the transmitted OAM beam is demultiplexed using an inverse lens for OAM mode *ℓ*_*n*_ = +1. The information on the two channels is then received by a dual-LP horn antenna and decoded, as shown in Fig. 4h, i. The two constellation diagrams exhibit excellent stability with minimal distortion, highlighting the system’s robust transmission performance across different polarization channels. Besides, the demultiplexing process completely reconstructs the two images, verifying the ability of the system to simultaneously transmit and recover independent data streams over dual-LP OAM channels.

The feasibility of implementing the OAM beam with frequency division multiplexing is subsequently verified. Inspired by the concept of asynchronous STM control, the entire metasurface is divided into two partitions, each operating at distinct STM frequencies, Δ*f*₁=100 kHz and Δ*f*₂=250 kHz. The details of the asynchronous STM control are presented in Supplementary Note S9. To maintain the symmetry of the vortex wavefronts, the two partitions are an inner square comprising 8 × 8 meta-atoms and an outer ring consisting of 80 meta-atoms, respectively. In the experimental setup, the feeding source emits monochromatic *x*-polarized waves at 26.8 GHz. Figure 4j shows that the DASM transmitter generates two OAM beams with mode *ℓ*_*n*_ = +1 and shifts their carrier frequencies to *f*₁ = 26.8001 GHz and *f*_2_ = 26.80025 GHz. These two distinct frequencies serve as independent communication channels, enabling simultaneous data transmission. Two distinct images are encoded into these channels, with both channels operating at a symbol rate of 50 kbps. The receiver is composed of a lens *ℓ*_*n*_ = +1 and a horn antenna detector. Figure 4k, l present the decoded constellation diagrams of the two channels. The QPSK constellation points are distinctly concentrated in four clusters, demonstrating notable signal quality and integrity. Meanwhile, the successfully recovered images transmitted through the two channels further validate the system’s ability to achieve frequency division multiplexing with negligible interference. These experimental results collectively highlight the practicality and efficiency of combining OAM-based communication with polarization or frequency division multiplexing.

### High-dimensional multiplexing transmitter

After confirming that OAM beams do not cause undesirable interference or coupling in current multiplexing techniques, we proceed to investigate integrating multiple multiplexing schemes to enable high-dimensional communication systems. As a proof-of-concept example, we establish a high-dimensional multiplexing transmitter employing three orthogonal physical dimensions for communications channel multiplexing: OAM mode division multiplexing, frequency division multiplexing, and polarization division multiplexing. Each of these dimensions provides two distinct states to encode information: the OAM dimension utilizes modes *ℓ*_1_ = -2 and *ℓ*_2_ = + 2, the polarization dimension adopts *x*- and *y*-polarizations, and the frequency dimension includes 26.8001 GHz and 26.80025 GHz. Note that higher OAM modes such as *ℓ*_*n*_ = ±3 cause higher crosstalk, as discussed in Supplementary Note S6. Hence, only OAM modes |*ℓ*_*n*_ | < 3 are implemented in the DASM-based transmitter. By combining these three independent dimensions, the system establishes a high-dimensional signal space with a total of 2 × 2 × 2 = 8 unique channels, significantly enhancing communication capacity.

**Fig. 5:**
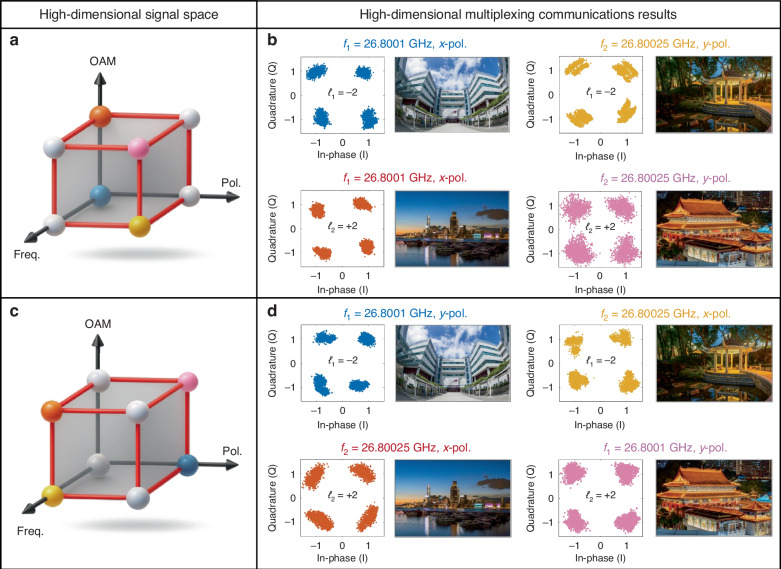
Measured high-dimensional multiplexing communication results. **a**, **c** Enabled communication channels of the high-dimensional signal space. The colorful balls represent the enabled channels, and the white balls represent the inactive ones. **b**, **d** Measured constellation diagrams and recovered images for the two subsets of channels, demonstrating robust communication performance of the high-dimensional signal space

However, due to the limitations of the experimental equipment, full 8-channel communication cannot be measured simultaneously. The control platform used in this demonstration is equipped with two PXIe-5783 cards (NI Corp.), each providing four pairs of I/O ports. Since two output ports are required to support a single QPSK data stream, the system can only accommodate 4 QPSK channels at a time. As a compromise, the high-dimensional signal space is divided into two subsets, as shown in Fig. 5a, c. Each subset is composed of 4 independent channels, which are transmitted simultaneously.

The transmitter setup for the two subsets generally aligns with the configuration used in the frequency division multiplexing experiments. The main difference is that both ports of the dual-LP source are activated for adding the polarization dimension. Moreover, the DASM synthesizes vortex beams carrying OAM modes *ℓ*_1_ = -2 and *ℓ*_2_ = + 2 through the implementation of specifically designed STM sequences. Figure 5a illustrates the first subset of the signal space, consisting of two vortex beams at *x*-polarization (26.8001 GHz) and *y*-polarization (26.80025 GHz). The colorful balls represent the enabled channels, while the white balls represent the inactive ones. At the receiver end, a pair of lenses is utilized to demultiplex the vortex beams and isolate the two corresponding OAM channels. A dual-LP horn is employed to detect the signals after removing the vortex. The measured constellation diagrams of the high-dimensional signal space are shown in the left panels of Fig. 5b. It can be observed that four distinct clusters are clearly identified, indicating good communication quality and minimal crosstalk among the four channels. Meanwhile, the high fidelity of the four recovered images shown in the right panel of Fig. 5b further indicates a low error rate and effective multi-channel communications. Using the same setup, the transmission of the remaining channels in the signal space is illustrated in Fig. 5c, and the received results of subset 2 are presented in Fig. 5d. The constellation diagrams and recovered images exhibit similar high-quality performance, reaffirming robust communication.

Therefore, the feasibility of enabling all eight channels is demonstrated by sequentially transmitting the two subsets, then combining the received data. Note that the lowest SNR of the eight channels is approximately 12.5 dB. According to Monte‑Carlo simulations for QPSK modulation under the same SNR condition, the corresponding bit‑error rate (BER) is around 10^-5^. In each communication channel, the transmitted image has a resolution of 360 × 240, i.e., 86,400 pixels. Each pixel is represented by 24 bits in RGB format, so that the total number of transmitted bits is 360 × 240 × 24 = 2.07 × 10^6^. With a BER of 10^-5^, the expected number of erroneous bits per image is therefore 2.07 × 10^6^ × 10^−5^ ≈ 21 bits, which corresponds to roughly one pixel with a slightly perturbed color value in each image. Consequently, all eight images are nearly 100% retrieved in the high-dimensional multiplexing framework. These results confirm that the proposed DASM can seamlessly combine OAM mode division with other multiplexing technologies, thus validating the high-dimensional multiplexing system.

## Discussion

In this work, we have demonstrated a high-dimensional multiplexing framework that integrates OAM-based communications with polarization and frequency division multiplexing. At the core of this high-dimensional multiplexing framework is DASM, specifically engineered to independently and precisely manipulate amplitude, phase, polarization, frequency, and OAM properties in real-time. We fabricated an FPGA-controlled DASM to establish the system, which is excited solely by a dual LP source without requiring external modulators. The communication data streams are encoded directly onto the OAM beams via STM sequences. Near-field measurements verify that the DASM is capable of generating a vortex beam carrying four distinct OAM modes. Furthermore, we showcase that the DASM enables the generation of 8 unrelated data streams through a single metasurface aperture by multiplexing across three orthogonal physical dimensions. The 8 channels are constructed based on two OAM modes, two polarizations, and two frequencies. Eight pictures are captured vividly at the receiver side, indicating low crosstalk among different channels.

Theoretically, our proposed high-dimensional multiplexing architecture can support an infinite number of communication channels, whereas the actual number of channels is subject to certain constraints in each dimension. For polarization, conventional polarization division multiplexing is fundamentally limited to at most three mutually orthogonal polarization channels, owing to the dimensionality of the Jones matrix formalism^55^. Therefore, only a small improvement can be achieved from the polarization dimension. The number of frequency channels in the DASM is determined by the number of partitions within the metasurface. Increasing the number of partitions allows more distinct modulation frequencies to be synthesized, thereby supporting more frequency channels. Meanwhile, the maximum number of usable OAM modes is restricted by the total number of meta‑atoms according to the Nyquist sampling theorem^56^. Higher‑order OAM modes exhibit more rapid azimuthal phase variations, which must be adequately sampled by the meta‑atoms to prevent spatial aliasing and excessive mode impurity. Hence, an effective method to scale the number of OAM channels is to incorporate more meta-atoms, such as by reducing meta-atom size or expanding the overall metasurface aperture. In addition, the increased number of meta-atoms also supports more independently controlled partitions, enabling more frequency channels.

The versatility of the DASM holds considerable promise for further enhancements. For example, expanding the present framework to incorporate extra multiplexing dimensions, such as time, spatial, or code division multiplexing, could unlock even higher communication dimensions. Allocating a subset of meta-atoms for direction-of-arrival estimation could seamlessly integrate sensing functionalities, thus endowing the framework with intelligent operations. Furthermore, the capability of synthesizing multiple OAM modes can be harnessed to implement quantum key distribution protocols, thereby enhancing the security of wireless communication networks. In summary, our proposed DASM establishes a flexible, adaptable, and software-defined foundation for high-dimensional multiplexing with a streamlined structure, offering a transformative solution for future high-capacity wireless communication frameworks.

## Methods

### DASM prototype design and fabrication

**Fig. 6:**
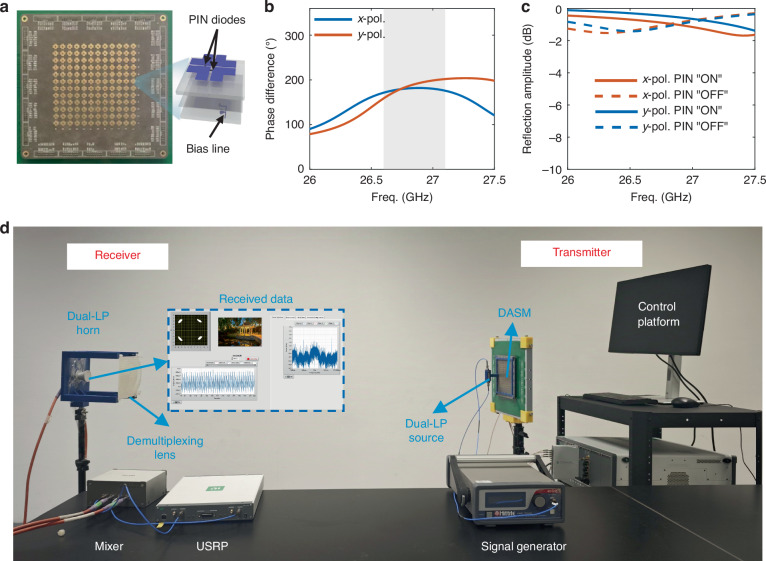
Details of the meta-atoms and the high-dimensional multiplexing communication experiment setup. **a** Fabricated DASM prototype and its meta-atom structure. **b** Measured phase difference between “ON” and “OFF” states for *x* and *y*-polarization. **c** Measured reflection amplitude of the meta-atom at “ON” and “OFF” states for *x* and *y*-polarization. **d** Experiments setup of the high-dimensional multiplexing communications

The proposed 12 × 12 meta-atoms DASM is fabricated using commercial printed circuit board technology, with PIN diodes mounted on its surface. Prototype photographs of the fabricated device are presented in Fig. 6a, while the configuration of its meta-atom is illustrated on the right (details see supplementary Fig. S12 and Table S4). The meta-atom is specifically designed to support dual-polarization operation at the center frequency of 26.8 GHz with a lattice size of 7 mm. It is composed of three metal layers, which are separated by two Rogers 5880 dielectric substrates characterized by a relative permittivity of 2.2 and a loss tangent of 0.0009. Each substrate has a uniform thickness of 0.508 mm. Four L-shaped metallic patches are symmetrically distributed on the top of the substrates. This symmetrical structure plays a critical role in precisely controlling the orthogonal LP EM waves and minimizing the cross-polarization, which is essential for achieving independent manipulation of the *x*- and *y*-polarization modes.

For the delivery of the FPGA output to toggle these diodes, the design includes careful arrangement of electrical connections: one L-patch is connected to the middle ground layer through a blind via hole, two L-patches on the two diagonals are connected to the bias lines on the third metal layer by through-holes, and the remaining patch serves to maintain the structural symmetry. Note that the fan-shaped sector introduced to the bias lines on the bottom metal layer is utilized as the RF choke, preventing unwanted RF currents from interfering with the bias circuitry.

The measured phase response of the meta-atom is presented in Fig. 6b. At 26.8 GHz, the meta-atom exhibits a phase difference close to 180° between the “ON” and “OFF” states of the PIN diodes for both *x*- and *y*-polarizations, proving its effective phase modulation performance. Figure 6c shows that the reflection loss of the meta‑atoms is better than −2 dB over the band of interest, indicating relatively low loss in the working frequency range. In addition, we define the operating bandwidth of the metasurface according to the phase response of the two switching states, i.e., the frequency range over which the phase difference between the ON and OFF states satisfies 180° ± 20°. Based on this criterion, the overlapping operating bandwidth of the proposed metasurface for both *x*- and *y*-polarizations is indicated by the gray area in Fig. 6b, spanning approximately 0.5 GHz from 26.6 to 27.1 GHz.

### High-dimensional multiplexing experiments setup

Figure 6c illustrates the indoor wireless communication experiment based on our DASM. The transmitter and receiver are aligned such that their broadside directions face each other. On the transmitter side, a microwave signal generator (AD HMC-T2240) is adopted to generate a monochromatic wave at 26.8 GHz. A 1-to-2 power divider is employed to split the monochromatic wave into two output signals with equal power and phase. For single-polarization experiments, one port of the 2 × 2 magnetoelectric dipole antenna array source is connected to the power divider, while the remaining port is terminated with a 50 Ω load. In dual-polarization cases, the two ports of the dual-polarized source are connected to the power divider. Two FPGA boards (Artix-7) implement STM sequences to each meta-atom of DASM for vortex beam synthesis. The FPGAs can receive data streams from the PXIe-5783 control platform and dynamically modulate the information carried by the vortex beams in a real-time manner. The modulated vortex beams with temporally variant phase information are directly produced and radiated into free space by the DASM.

At the receiver side, a dual-LP horn antenna is placed behind an OAM demultiplexing lens. After removing the vortex, the received signal from the horn is down-converted by a mixer (TMYTEK UD Box 5 G) from the millimeter-wave range to lower than 6 GHz. The converted low-frequency signal is then received and decoded by a software-defined radio transceiver (NI USRP 2944 R).

The transmission distance between the DASM aperture and the receiver aperture in communications experiments is 0.4 m. To figure out the relationship between communication performance and transmission distance, we measured the signal-to-noise ratio (SNR) versus transmission distance for one channel configuration (*ℓ*_*n*_ = + 1, *x*-polarization and 26.8001 GHz). It can be observed in Supplementary Fig. S13 that SNR drops rapidly as the transmission distance increases, which is mainly due to the divergent nature of OAM beams and the large free‑space path loss at millimeter‑wave frequencies. The far‑field boundary of our DASM is approximately 1.26 m, where the SNR falls to ~3 dB. As a result, the demonstrated OAM communication system is suitable for near‑field applications, such as wireless power transfer^57^, intra-device communications^58^, and data center interconnections ^59^.

## Supplementary information

Supplementary

## Data Availability

The data that support the findings of this study are available from the corresponding author upon reasonable request.

## Code availability

The codes that support the theoretical modeling of the metasurfaces are available from the corresponding authors upon reasonable request.
