# Supplementary material for: High-dimensional multiplexing through vortex electromagnetic wave manipulation by space-time-coding metasurfaces
Source: Light Sci Appl. 2026 Mar 9;15:160. doi: 10.1038/s41377-026-02232-6 (PMC12968085; doi:10.1038/s41377-026-02232-6)
Supplement: Supplementary file 1 — Supplementary [file 41377_2026_2232_MOESM1_ESM.docx]

**Supplementary Information for**

**High-dimensional multiplexing through vortex electromagnetic wave manipulation by space–time–coding metasurfaces**

Chenfeng Yang^1,2,†^, Si Ran Wang^1,2,†^, Jia Chen Du^1,2^, Man Ting Wang^3^, Zheng Xing Wang^1,2^, Ka Fai Chan^1^, Dongze Zheng^4^, and Geng-Bo Wu^1,2*^

*^1^State Key Laboratory of Terahertz and Millimeter Wave, City University of Hong Kong, Hong Kong, 999077, China*

*^2^Department of Electrical Engineering, City University of Hong Kong, Hong Kong, 999077, China*

*^3^Department of Electrical and Computer Engineering, University of Alberta, Edmonton, AB T6G 2R3, Canada.*

*^4^School of Electronics Science and Engineering, University of Electronics Science and Technology of China, Chengdu, 611731, China.*

^†^These authors contributed equally to this work

^*^Corresponding author. Email: [bogwu2@cityu.edu.hk](mailto:bogwu2@cityu.edu.hk)

**This Supplementary Information includes:**

Supplementary Notes S1 to S9

Supplementary Figures S1 to S13

Supplementary Equations S1 to S9

Supplementary Tables S1 to S4

**Supplementary Note S1: Independent phase and amplitude control through spatial-temporal modulation**

Each polarization of the proposed meta-atom is loaded with a single PIN diode. For time-invariant control, the reflection state of the meta-atom can be toggled between “ON” and “OFF” by adjusting the bias voltage applied to the PIN diode. It is important to note that the phase control is discrete rather than continuous, and the reflection amplitude remains fixed. By introducing temporal modulation, independent and continuous phase and amplitude control can be achieved at the harmonic frequencies. Changing the static bias voltage to a time-varying sequence with the periodicity of *T*_0_ = 1/*f*_0_, the STCM can transform an incident monochromatic wave at frequency *f_c_* into multiple discrete harmonics. These generated harmonics are evenly distributed in the frequency domain around the carrier frequency *f_c_* with a frequency separation of *f*_0_.

Suppose “ON” and “OFF” states of the meta-atom are represented by |*Γ*_1_|*e^jφ^*^1^ and |*Γ*_2_|*e^jφ^*^2^, respectively, where *Γ*_1_ and *Γ*_2_ denote the reflection magnitudes, as well as *φ*_1_ and *φ*_2_ imply the reflection phases of the two states. Accordingly, the time-varying reflection coefficient can be expressed as:

$$\begin{aligned} \Gamma(t)=\left\{ \begin{aligned} &\left| \Gamma_{1} \right|\cdot e^{j\varphi_{1}}, mT_{0}<t<mT_{0}+\Delta t \text{or }mT_{0}+\Delta t+\tau<t<(m+1)T_{0} \\ &\left| \Gamma_{2} \right|\cdot e^{j\varphi_{2}}, mT_{0}+\Delta t\leq t\leq mT_{0}+\Delta t+\tau\end{aligned} \right.\#\left( S1 \right) \end{aligned}$$

Taking the Fourier series expansion of the reflection coefficient Eq. (S1), the *k*th-order harmonic component is expressed as follows:

$$\begin{aligned} a_{k}=\left\{ \begin{aligned} M\left| \Gamma_{1} \right|e^{j\varphi_{1}}+\left( 1-M \right)\left| \Gamma_{2} \right|e^{j\varphi_{2}}, k=0 \\ &\left( \left| \Gamma_{1} \right|e^{j\varphi_{1}}-\left| \Gamma_{2} \right|e^{j\varphi_{2}} \right)M\cdot|Sa(k\pi M){|e}^{-j\left\{ k\omega_{0}\Delta t+\frac{\pi}{2}\left[ 1-\left( -1 \right)^{\left\lfloor\left| k \right|\cdot M \right\rfloor} \right] \right\}}, k=\pm1,\pm2,\pm3,\ldots\end{aligned} \right.\#\left( S2 \right) \end{aligned}$$

where Δ*t* is the time delay, ⌊·⌋ represents the rounding down function, *M* is the duty ratio equal to *τ/T*_0_, and *Sa*(·) represents sin(*kπM*)/*kπM*. Without loss of generality, the reflection magnitudes are set equal *Γ*_1_ = *Γ*_2_ = 1, with phase values *φ*_1_ = π and *φ*_2_ = 0. Figure S1a shows the reflection coefficient in one period, where the pulse has a duty ratio *M* and time delay *Δt*. In the frequency domain, the coefficient of higher-order harmonics can be derived as:

$$\begin{aligned} a_{k}=\text{2}M\cdot|Sa\left( k\pi M \right){|e}^{-j\left\{ k\omega_{0}\Delta t+\frac{\pi}{2}\left[ 1-\left( -1 \right)^{\left\lfloor\left| k \right|\cdot M \right\rfloor} \right] \right\}}, k\neq0\#\left( S3 \right) \end{aligned}$$

The amplitude *A* and the phase *φ* of the +1st harmonic reflected waves can be calculated from Eq. (S3) as follows:

$$\begin{aligned} A=2M\left| Sa\left( \pi M \right) \right|, 0\leq M\leq\frac{1}{2} \#\left( S4 \right)\# \end{aligned}$$

$$\begin{aligned} \varphi=-\omega_{0}\Delta t, 0\leq\Delta t<T_{0}\#\left( S5 \right) \end{aligned}$$

According to Eqs. (S4) and (S5), the amplitude and phase of the harmonics are governed by the duty ratio *M* and the time delay Δ*t*, respectively. In the FPGA output logic, one modulation period is divided into multiple time slots, each assigned a value of either 0 or 1. This output logic makes sure that both the time delay and the duty ratio of the pulse can be flexibly and independently controlled if the number of time slots in one period is sufficiently large. Figures S1b and S1c depict the phase and amplitude responses as Δ*t* and *M* vary. It can be observed that the phase can be continuously controlled over a full 2π range without affecting the amplitude by changing Δ*t* from 0 to *T*_0_. For the amplitude modulation process, the amplitude initially increases with *M*, reaching the maximum at *M*=0.5, before decreasing. Notably, the phase remains constant throughout the amplitude modulation process. These results indicate that the complete amplitude and phase characteristics of the harmonics can be controlled by the temporal modulation parameters of the applied bias voltage.


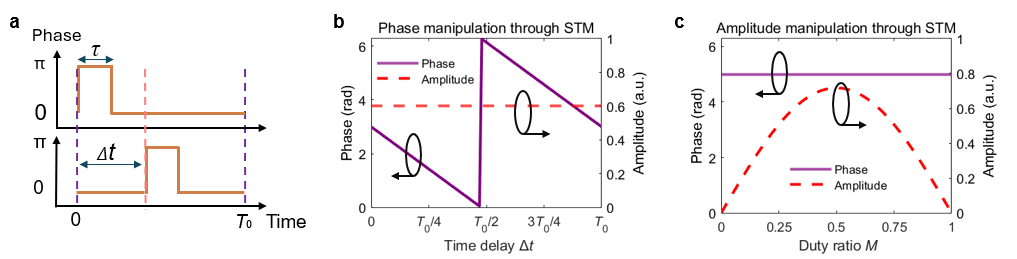


**Supplementary Figure S1 | Independent and continuous control of amplitude and phase through STM. a** Illustration of one period of the time-varying reflection coefficients. **b** Phase manipulation of the +1st harmonic by varying Δ*t*. **c** Amplitude manipulation of the +1st harmonic by varying *M.*

**Supplementary Note S2: Dual-LP source design and fabrication**

The employed source in the experiment is a 2×2 element dual-LP magnetoelectric (ME) dipole antenna array. It is fabricated using printed circuit board techniques, and a prototype is shown in Figure S2a. As shown in Figure S2b, each ME dipole antenna is composed of four leaf-shaped patches and two L-shaped probes for dual-LP operation. Figure S2c shows that the whole ME dipole antenna has three substrate layers. The two L-shaped probes are located in different layers to ensure high isolation between the two ports. The leaf-shaped patches improve impedance matching by shortening current paths while maintaining the patch geometry, resulting in a wide operating bandwidth.

Four ME-dipole antennas are arranged into an array to improve gain performance. A T-junction power divider feeding network ensures uniform power distribution and in-phase excitation of the 2×2-element array. The simulated and measured performance of the dual-LP source antenna is shown in Figure S3. It can be observed that the reflection coefficients for both LPs are lower than -10 dB from 20 GHz to 28 GHz, with isolation less than -20 dB. Due to the symmetric design for both polarizations, the gain results in Figure S3d are nearly identical. The *x*-polarization feed line is located on the bottom layer of substrate-I, which leads to the radiation energy propagating through the substrate. This structure results in a slight difference in gain performance between *x*- and *y*-polarizations. Nevertheless, the results still demonstrate satisfactory radiation performance of the employed source for both polarizations.


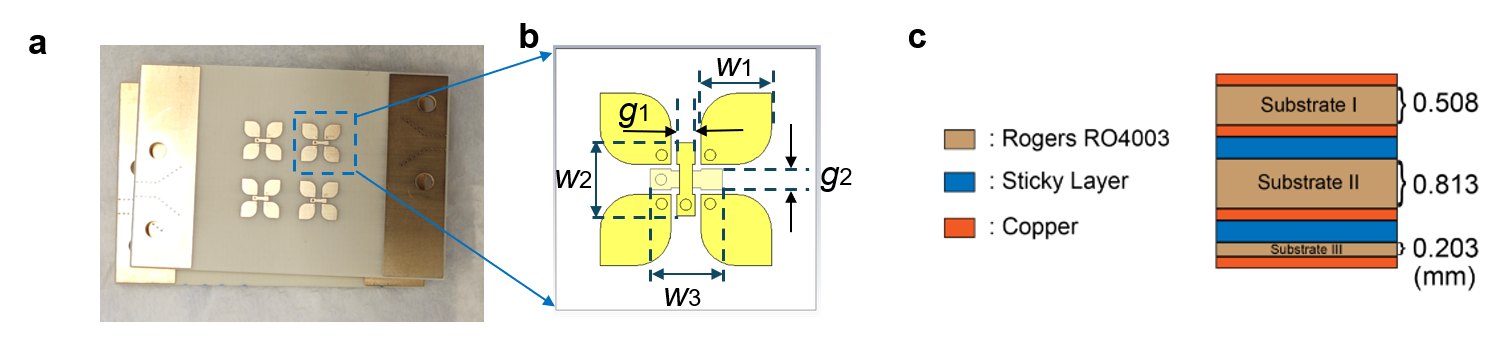


**Supplementary Figure S2 | Structure of the dual-LP source. a** Photos of the employed dual-LP source. **b** Top view of a ME-dipole antenna. **c** Side view of the employed source*.*

**Table S1 | Dimensions of the dual-LP ME dipole**

| Parameters | ***w*_1_** | ***w*_2_** | ***w*_3_** | ***g*_1_** | ***g*_2_** |
| --- | --- | --- | --- | --- | --- |
| Value (mm) | 1.9 | 1.95 | 1.95 | 0.5 | 0.55 |


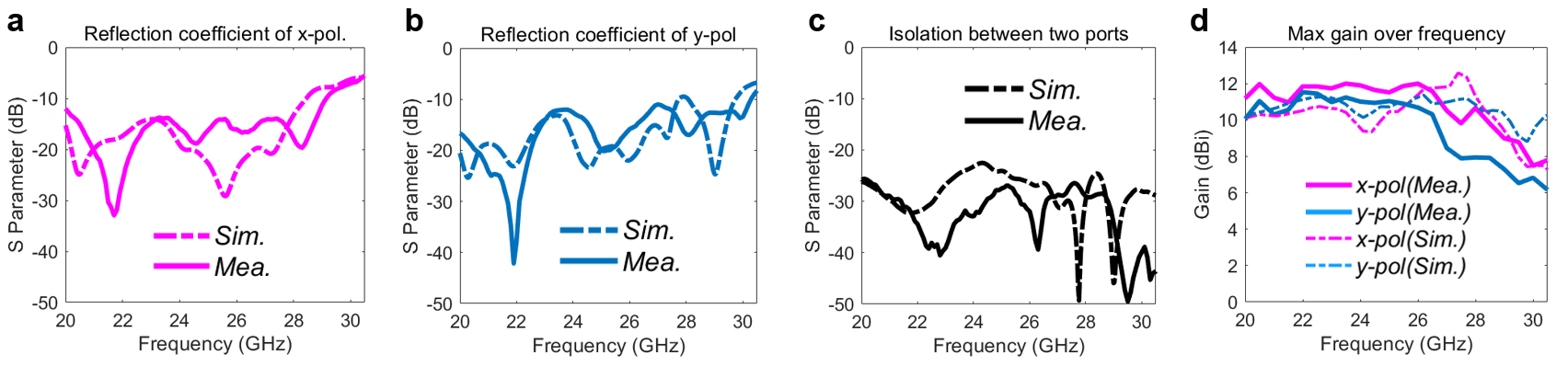


**Supplementary Figure S3 | Simulated and measured performance of the dual-LP source. a** Port reflection coefficient of the source at *x*-polarization. **b** Port reflection coefficient of the source at *y*-polarization. **c** Isolation of the source between the two ports*.* **d** Source gain responses.

**Supplementary Note S3: Near-field experiments setup and results**

Figure S4 displays the STCM prototype and its measurement setup. The near-field measurement system is built in a microwave chamber to evaluate the OAM generation performance of the STCM. In the experimental configuration, the K-band horn antenna is tilted at a 45° angle and positioned at coordinates (42, 0, 42) mm, with the coordinate origin located at the center of the metasurface. The horn is connected to a microwave signal generator (HMC-T2240) to produce 26.8 GHz momochromatic waves for exciting the STCM. To capture the reflected E-fields, a receiving probe connected to the vector network analyzer (Keysight N5242B) is employed. The probe is capable of detecting a wide range of frequencies, including the harmonics produced by the STCM.

One challenge in this measurement is the difficulty in obtaining an accurate spatial phase distribution, since the incident source and the receiving probe operate at different frequencies. To overcome this, a reference antenna is placed above the STCM, operating at the same frequencies as the generated harmonics to provide a phase reference^1^. The position of the reference antenna is fixed as the probe moves over the sampling plane. Both the reference antenna and the scanning probe are connected to the same vector network analyzer. The near-field amplitude and phase distributions are extracted by comparing the received signals of the scanning probe and the reference antenna.


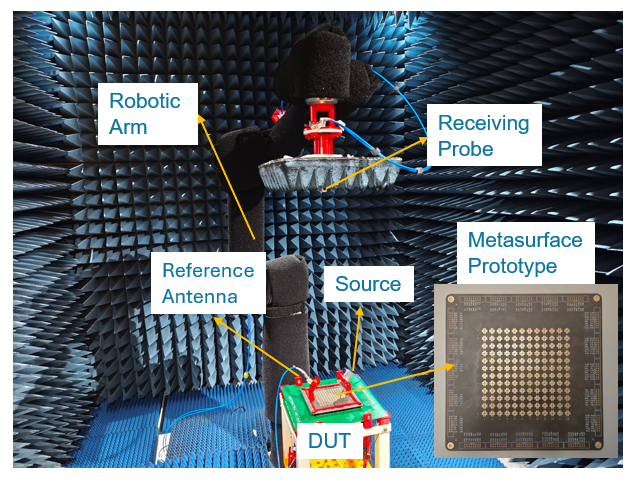


**Supplementary Figure S4 | Photograph of the near-field experiments setup.**

To obtain the planar E-field distribution, the receiving probe is attached to a robotic arm, allowing free movement within a 3D space. The E-field in the near-field region is measured on a transverse plane 81 mm from the metasurface aperture, which covers a square of 70 mm by 70 mm. The measurement plane is sampled with a spatial step size of 2.33 mm, ensuring sufficient resolution to capture detailed field variations. The resulting near-field amplitude distribution, obtained from this setup, is presented in Figure S5.


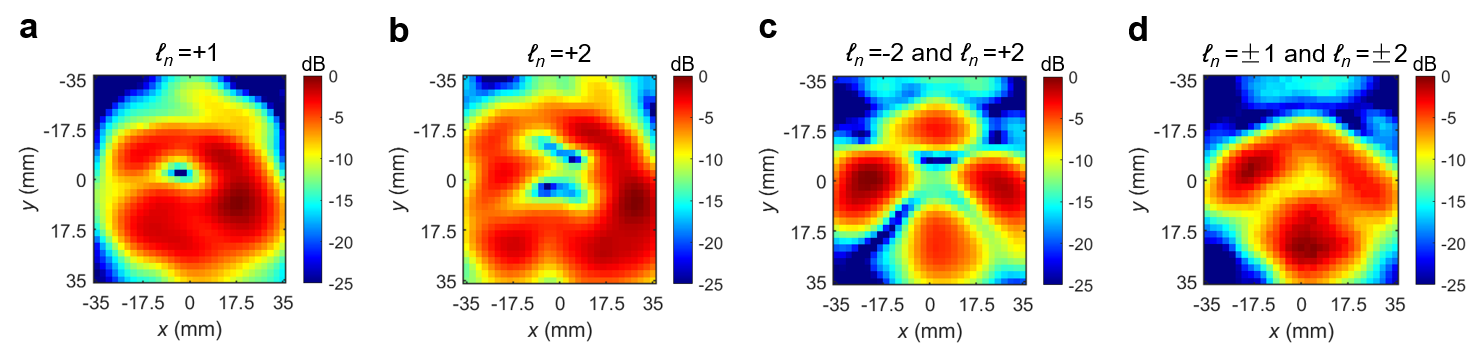


**Supplementary Figure S5 | Measured near-field amplitude distribution of the generated vortex beam. a** OAM mode *ℓ_n_*=+1. **b** OAM mode *ℓ_n_*=+2. **c** OAM mode *ℓ_n_*=±2*.* **d** OAM mode *ℓ_n_*=±1, ±2.

**Supplementary Note S4: Mode purity of the generated vortex beam**

To analyze OAM purity, the radiated field of the OAM generator can be decomposed into a superposition of a series of integer OAM modes, i.e., OAM spectrum, which is obtained by a Fourier transform of the field distribution E(*φ*) as follows:

$$\begin{aligned} P(\mathcal{l}_{n})=\frac{1}{\sqrt{2\pi}}\int_{0}^{\infty} \int_{0}^{2\pi} E(\varphi)e^{-j\mathcal{l}_{n}\varphi}d\varphi dr\#\left( S6 \right) \end{aligned}$$

Then, the mode purity of each OAM mode can be calculated as follows:

$$\begin{aligned} \eta\left( \mathcal{l}_{n} \right)=\frac{P\left( \mathcal{l}_{n} \right)}{\sum_{q=-\infty}^{\infty} P\left( \mathcal{l}_{q} \right)} \#\left( S7 \right)\# \end{aligned}$$

Figure. S6 displays the calculated OAM mode purity spectrum using the measured results. For a single-mode OAM beam in Figures S6a and S6b, the mode purity exceeds 90%. When generating multiple OAM beams, the expected modes dominate the spectrum, as shown in Figures S6c and S6d. All of these results have successfully demonstrated the capability of the proposed DASM to generate multiple OAM modes through a single antenna aperture.


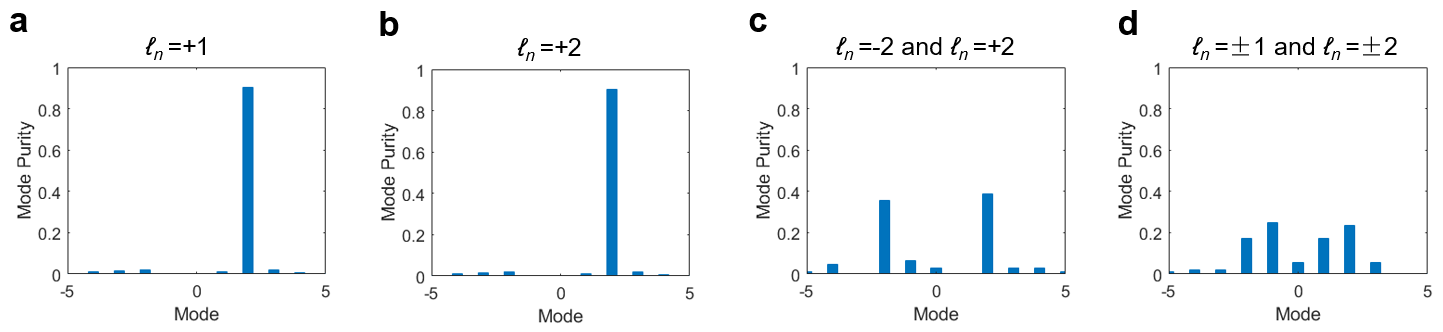


**Supplementary Figure S6 | Measured mode purity spectrum of the generated vortex beam. a** OAM mode *ℓ_n_*=+1. **b** OAM mode *ℓ_n_*=+2. **c** OAM mode *ℓ_n_*=±2*.* **d** OAM mode *ℓ_n_*=±1, ±2.

**Supplementary Note S5: Design of the OAM demultiplexer**

The OAM beam propagating in the free space can be described as a Gaussian beam modulated with a helical phase factor exp(*iℓ_n_ϕ*), where *ℓ_n_* is the mode number and *ϕ* is the azimuthal angle. The unique twisted wavefront is the basis of OAM-based multiplexing. Applying an inverse phase mask exp(-*iℓ_n_ϕ*) can remove the vortex and convert the OAM beam back to a standard Gaussian beam. For multiplexing *m* independent OAM channels, the total E-field in the free space is the summation of all the modulated beams^2^:

$$\begin{aligned} E_{\mathrm{MUX}}\left( r,t,\phi\right)=\sum_{n=1}^{m} S_{n}\left( t \right)\cdot exp\left( i\mathcal{l}_{n}\phi\right)\cdot Mag\left( r \right)\#\left( S8 \right) \end{aligned}$$

where *S_n_*(*t*) is the data information encoded to OAM mode *ℓ_n_*, *Mag*(*r*) represents a function of the complex E-field amplitude at the waist of the Gaussian beam, and *r* denotes the radial distance from the center axis of the beam. Note that during propagation through free space, the momentum and information encoded in each OAM mode remain unchanged. For information demultiplexing, an inverse phase mask exp(-*iℓ_k_ϕ*) is able to transform the superposed OAM beams as follows:

$$\begin{aligned} E_{\mathrm{DEMUX}}\left( r,t,\phi\right)&=\exp\left( -i\mathcal{l}_{k}\phi\right)\sum_{n=1}^{m} S_{n}\left( t \right)\cdot exp\left( i\mathcal{l}_{n}\phi\right)\cdot Mag\left( r \right) \\ &=S_{k}\left( t \right)\cdot Mag\left( r \right)+\sum_{n=1,n\neq k}^{m} S_{n}\left( t \right)\cdot exp[i\mathcal{(l}_{n}-\mathcal{l}_{k})\phi]\cdot Mag\left( r \right)\#\left( S9 \right) \end{aligned}$$

From Eq. (S9), the demultiplexed E-field is decomposed into two terms: the first term corresponds to the Gaussian beam, and the second term to the OAM beam. In the first term, the OAM mode *ℓ_k_* with phase component exp(-*iℓ_k_ϕ*) is removed, and the information *S_k_*(*t*) can be easily obtained by a conventional horn antenna. The second term consists of the remaining OAM modes, which retain the inherent doughnut-shaped intensity profiles. There is a distinct null in intensity at the center of the doughnut. Due to this center null, minimal energy from the second component can be detected, making it difficult to retrieve the information carried by these residual OAM modes. Consequently, only the corresponding OAM channel’s information is effectively extracted by the inverse phase demultiplexer.


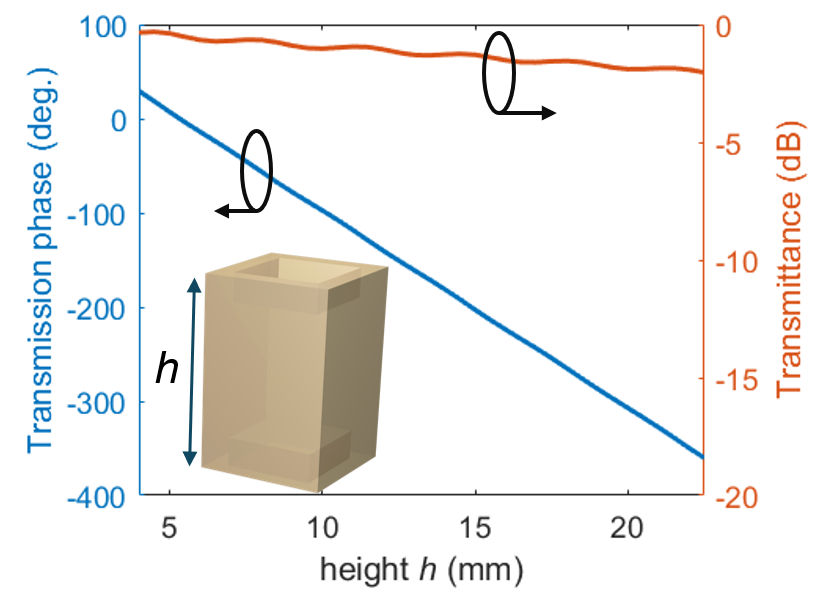


**Supplementary Figure S7 | OAM demultiplexer element structure and its simulation performance**.

A discrete dielectric lens (DDL) structure composed of an array of elements is employed for designing the demultiplexer with inverse spiral phase distribution. The DDL can be manufactured by a low-cost and fast 3D-printing method. The commercial EM simulator software ANSYS HFSS is used to design the element made of resin material (dielectric constant *ε_r_* = 2.77 and loss tangent *σ* = 0.02). As shown in Figure S7, the height of the element manipulates its transmission phase, which covers a full 360° range. Meanwhile, a high transmission amplitude (>-2dB) is maintained across the range of height variations. By engineering a helical phase distribution across the lens surface, the lens can convert the OAM beam back into a fundamental Gaussian beam. In this paper, six lenses have been fabricated, each capable of demultiplexing one of six distinct OAM channels, and photos of the fabricated prototypes are presented in Figure S8.


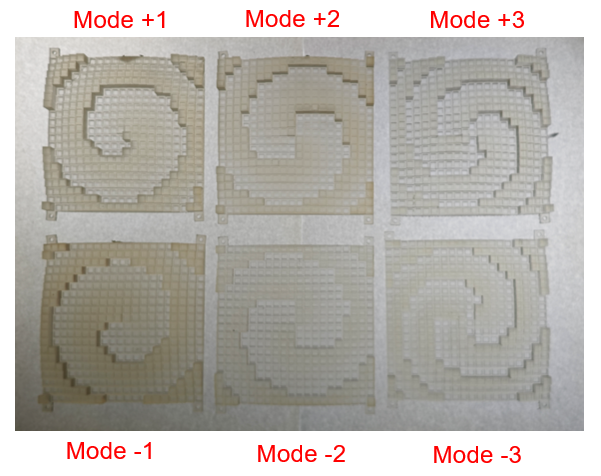


**Supplementary Figure S8 | Photos of six OAM demultiplexing lenses.**

**Supplementary Note S6: Influence of higher OAM modes.**

Our proposed OAM multiplexing strategy is not restricted to *ℓ_n_* = ±1 and ±2 and can be extended to higher-order OAM modes. However, the quality of higher-order OAM channels (e.g., inter-channel crosstalk) is degraded gradually as |*ℓ_n_*| increases. The limited OAM channels can be attributed to several practical factors: finite transmitter/reciever aperture size, increased beam divergence and sensitivity to misalignment. To further clarify the influence of higher‑order OAM modes, we experimentally measured the crosstalk for different sets of OAM channels.

During multiple OAM channels demultiplexing, crosstalks always exist because the demultiplexed field generally contains one desired Gaussian beam mode and several residual OAM modes from other channels. Only the Gaussian beam component is useful for information retrieval, while the remaining OAM components are regarded as crosstalk^3^. In our work, the crosstalk of each OAM channel is quantified by comparing the interference power coupled from all other channels with the desired power of the target channel itself. For a transmitted OAM beam with mode *ℓ_n_* and a demultiplexing receiver with mode *ℓ_k_*, we define 𝑃*_k=n_* as the target received power, and 𝑃*_k≠n_* as the sum of the interference power when demultiplexing OAM modes are *k≠n*. The crosstalk of OAM channel *ℓ_n_* is then calculated as *XT_n_*=10*log_10_(𝑃*_k≠n_* / 𝑃*_k=n_*).

Tables S2 and S3 summarize the measured crosstalk for OAM modes from −2 to +2 and from −3 to +3, respectively. It can be observed that the crosstalk of all channels increases when higher‑order OAM modes are added to the system. The stronger mutual interference can be attributed to the fact that more OAM channels are accommodated within the same physical aperture, which reduces the power allocated to each individual OAM channel. Moreover, the crosstalk of the higher‑order OAM modes ±3 is larger than that of the lower‑order modes in Table S3. This is because the higher‑order OAM beams are more divergent, and less target power can be captured by the receiver with a fixed aperture size. To ensure the stability of the wireless communications, we demonstrate the high-dimensional multiplexing framework by using limited OAM modes |*ℓ_n_*|<3.

Several methods can be applied to reduce crosstalk and increase the number of achievable OAM channels. On the receiver side, enlarging the effective aperture or shortening the transmission distance allows more power from higher‑order OAM modes to be collected, thereby improving the target received power. On the transmitter side, increasing the number of meta-atoms on metasurfaces (i.e., reduce the size of meta‑atoms) enhances the spatial resolution of the aperture field, which improves the mode purity of each synthesized OAM beam and mitigates crosstalk among different channels.

**Table S3 | The measured cross talk for OAM channels with *ℓ_n_*= ±1, ±2, and ±3**

| *ℓ_n_* | -3 | -2 | -1 | 1 | 2 | 3 |
| --- | --- | --- | --- | --- | --- | --- |
| **Crosstalk (dB)** | **-5.6** | **-8.9** | **-11.0** | **-10.1** | **-7.9** | **-5.2** |

**Table S2 | The measured cross talk for OAM channels with *ℓ_n_*= ±1 and ±2**

| *ℓ_n_* | -2 | -1 | 1 | 2 |
| --- | --- | --- | --- | --- |
| **Crosstalk (dB)** | **-11.2** | **-11.8** | **-10.9** | **-9.9** |

**Supplementary Note S7: Design of a metasurface-based receiver for simultaneously demultiplexing all OAM channels.**

In practical applications, the lens-based recievers are not efficient to demultiplex OAM beams simultaneously. However, the demultiplexing lens is no longer necessary if the complex E‑field distribution at the receiver aperture can be measured. Instead, we can numerically apply an inverse phase mask of the form exp(-*iℓ_k_ϕ*) in post‑processing. According to Eq. (S7), this operation yields two terms: a Gaussian‑beam term and an OAM‑beam term. Owing to the orthogonality of OAM modes, only the Gaussian‑beam term contributes when we integrate the demultiplexed E‑field over the aperture. The OAM‑beam terms vanish because their angular dependence exp[*i*(*ℓ_n_*-*ℓ_k_*) *ϕ*] (*n*≠*k*) integrates to zero over *ϕ.* Consequently, if the aperture E‑field distribution is known, one can recover the information carried by each OAM channel simply through applying the corresponding inverse phase mask and performing the aperture integral.

The problem of characterizing the EM response at each spatial position across an aperture remains an active and important research topic. For example, an adaptive programmable meta-atom equipped with sensing circuits is proposed to retrieve the local E‑field distribution over the metasurface^4^. However, integrating sensing circuits into every meta‑atom is both expensive and complex in practice. A low-cost and efficient approach is to use specific coding sequences to reconstruct the E‑field using a single receiver^5^. Although the coding scheme is used to detect the direction of arrival of EM waves^5^, the same principle can be extended to retrieve the phase and amplitude distribution of OAM beams. Specifically, the reflection states of all meta‑atoms are arranged to form a Hadamard matrix, whose rows are mutually orthogonal and therefore invertible. Under OAM illumination, the contributions from all meta‑atoms are superimposed in space and captured by a single receiving horn antenna as a one‑dimensional time signal. By collecting this received signal over the full coding period and using the Hadamard matrix as a key, the incident E-field on every meta‑atom can be mathematically derived. Afterwards, the information of each OAM channel can be decoded simultaneously by applying numerical phase mask post‑processing and performing aperture integration as described above.

**Supplementary Note S8: Limitations of the modulation frequencies and possible solutions**

The limitations of the transmission symbol rate in our experiment mainly come from the hardware implementation rather than the DASM concept itself. In our system, information is loaded onto the communication channels by STM sequences of PIN-diode-loaded meta‑atoms, which are driven by an FPGA. The switching time of the MACOM MADP‑000907‑14020x PIN diodes is about 2–3 ns, and the Artix‑7 FPGA supports a maximum clock frequency of 628 MHz. Therefore, the metasurface could, in principle, support modulation of the sub-GHz range or even higher.

The main bottleneck in the current prototype arises from the process of using an FPGA to control DASM. The low-speed interconnects, the non-optimized PCB routing, the low-performance regulator, and the voltage transformer chips all introduce additional delay and distortion into the control signals. Figure S8a shows a photograph of the connection between the FPGA control board and the DASM. This imperfect board‑to‑board connection introduces parasitic inductance and capacitance, whose impact becomes increasingly significant at higher frequencies. Figures S8b, S8c, and S8d present the measured output waveforms of the FPGA control board at different switching frequencies. Although some ripples appear at 10 MHz, the voltage level remains higher than 1.33 V (turn-on voltage of the PIN diode), allowing the 10 MHz FPGA output to reliably drive the PIN diodes in our DASM system. However, the FPGA output waveforms suffer from severe distortion at a higher control frequency of 50 MHz. The distortion degrades the effective STM sequence on the metasurface and leads to unstable communication performance. To guarantee nearly square waveforms for this proof-of-concept demonstration, we intentionally limited the switching speed of the PIN diodes to 10 MHz.


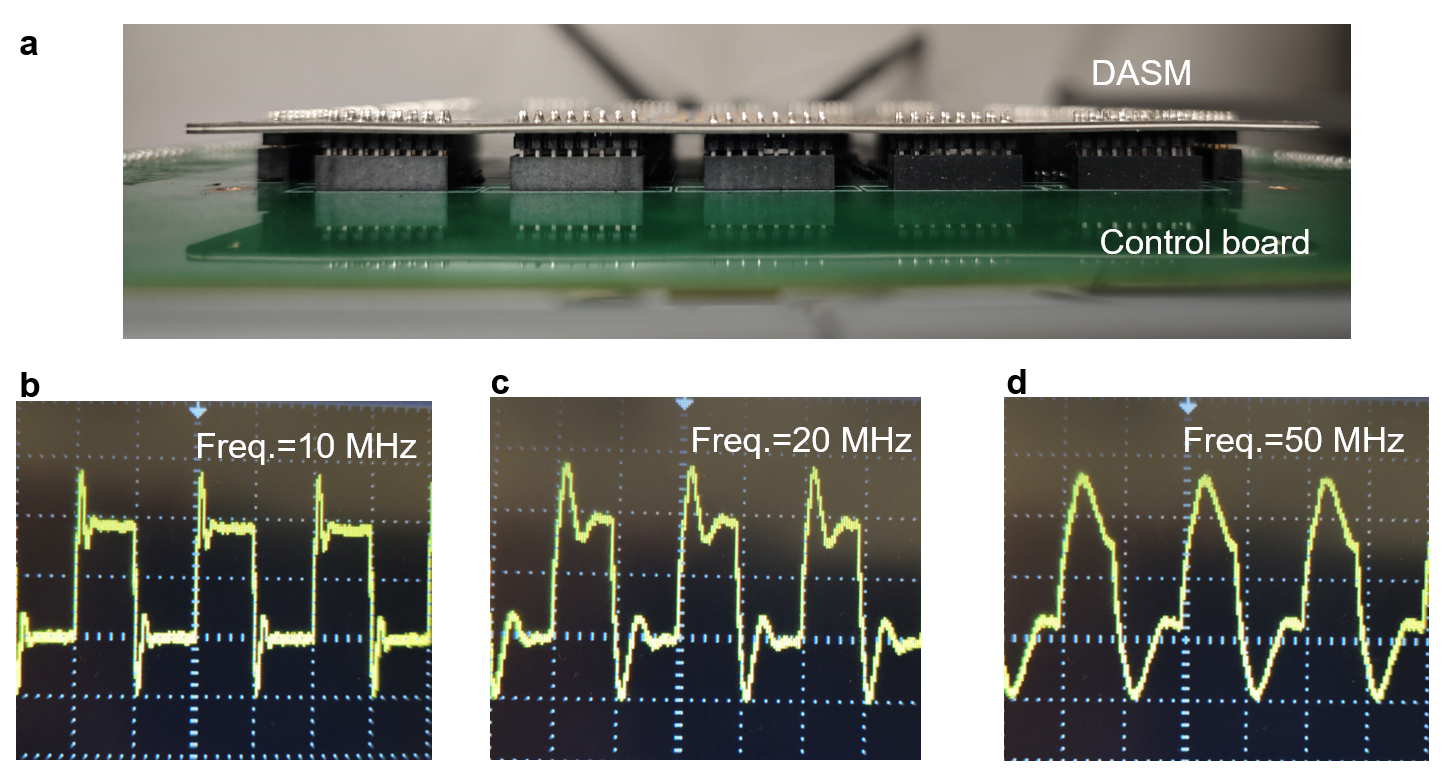


**Supplementary Figure S9|** **a** Photographs of the interconnect between the FPGA control board and metasurface board. **b-d** Measured control signal waveform from the control board output with switching frequencies of 10MHz, 20MHz, and 50MHz.

According to Supplementary Note S1, the phase manipulation ability in the STM scheme depends on the phase delay *Δt* within one modulation period *T*_0_. The switching speed of meta-atoms’ states determines the minimum achievable *Δt*. To realize quasi-continuous phase control for high-quality OAM beam generation, we choose *T*_0_ = 100*min(*Δt*), which yields a phase resolution of 360°/100 = 3.6°. With a 10 MHz PIN‑diode switching rate 1/min(*Δt*), the modulation frequency *Δf* is 10 MHz / 100 = 100 kHz. For QPSK data transmission, the symbol rate must be lower than *Δf* to maintain robust demodulation. Therefore, we set the symbol rate to 50 kbps, i.e., half of *f*_0_, which provides sufficient margin for practical communications.

The current limitation on the transmission data rate can be mitigated by adopting more advanced digital‑control solutions. For example, using high‑speed interconnects (such as PCI Express) and high-performance electronic components can directly improve the quality of the digital process. It is also possible to co‑design the FPGA, or a dedicated driver ASIC, together with the metasurface on the same PCB to eliminate long interconnections. During PCB layout, employing high‑speed routing strategies and proper impedance matching will further reduce the distortion of control signals. Correspondingly, the achievable symbol rate could be scaled to Mbps, which would be more consistent with the high carrier frequency at the millimeter-wave band.

Although metasurfaces have been thoroughly investigated from the microwave to the optical regimes, the PIN diodes used in this work are usable only up to about 70 GHz. To further increase the symbol rate, one promising route is to extend the operating frequency of the DASM platform to higher bands, thereby providing a larger available bandwidth. Achieving such frequency scaling requires suitable high‑speed active tuning mechanisms. In the terahertz range, the required temporal modulation can be implemented using advanced electronic components, such as CMOS chips^6^ and molybdenum disulfide-based devices^7^. For operation in the optical regime, phase‑change material such as Ge_2_Sb_2_Se_4_Te^8^ can be employed as switches based on the distinct optical responses between amorphous and crystalline phases. By integrating these switching elements into the metasurface, the proposed DASM‑based multiplexing framework can be extended to much higher frequency bands and provide higher communication symbol rates.

**Supplementary Note S9: Details of asynchronous** **spatial-temporal modulation.**

The frequency domain of the high-dimensional multiplexing is achieved by asynchronous STM. As proof of concept, two frequency values are chosen for frequency division multiplexing. The metasurface is conceptually divided into two partitions, each driven by separate modulation frequencies Δ*f*₁ and Δ*f*_2_. The reflection coefficients for the two input time sequences are shown in the left panel of Figure S8. The duty cycles and time delays of the two sequences are completely independent of each other. Under the illumination of a monochromatic incidence *f*_0_, two series of harmonics are generated by the ASTCM (*f*_1_ = *f*_0_ +*m*Δ*f*₁, and *f*_2_ = *f*_0_ + *n*Δ*f*_2_, where *m* and *n* are integers). However, the EM properties among different harmonics are entangled in one series according to Eq. (4) in the main text. Specifically, the phase and amplitude of the harmonics in series #1 depend on sequence #1, while those in series #2 also depend on sequence #2. For a given symbol rate *Rs*, the main spectral components of each QPSK band are confined approximately within [*f*_1_ -*Rs*/2, *f*_1_ +*Rs*/2] and [*f*_2_ -*Rs*/2, *f*_2_ +*Rs*/2]. The two bands should not overlap in order to avoid mutual interference between the frequency channels.

In the experiments, the incident wave frequency is 26.8 GHz, and the modulation frequencies of the two regions are Δ*f*₁ = 250 kHz and Δ*f*_2_ = 100 kHz. Taking *m* = *n* = 1 as an example, the corresponding operating frequencies of the ASTCM are  *f*_1_ = 26.80025GHz and *f*_2_ = 26.8001GHz. By independently adjusting the duty cycles and time delays of the two sequences, different phase and amplitude values of the two harmonics can be synthesized, analogous to the phasor representation illustrated in the right panel of Figure S10. Therefore, frequency-division multiplexing can be achieved using the asynchronous STM strategy.


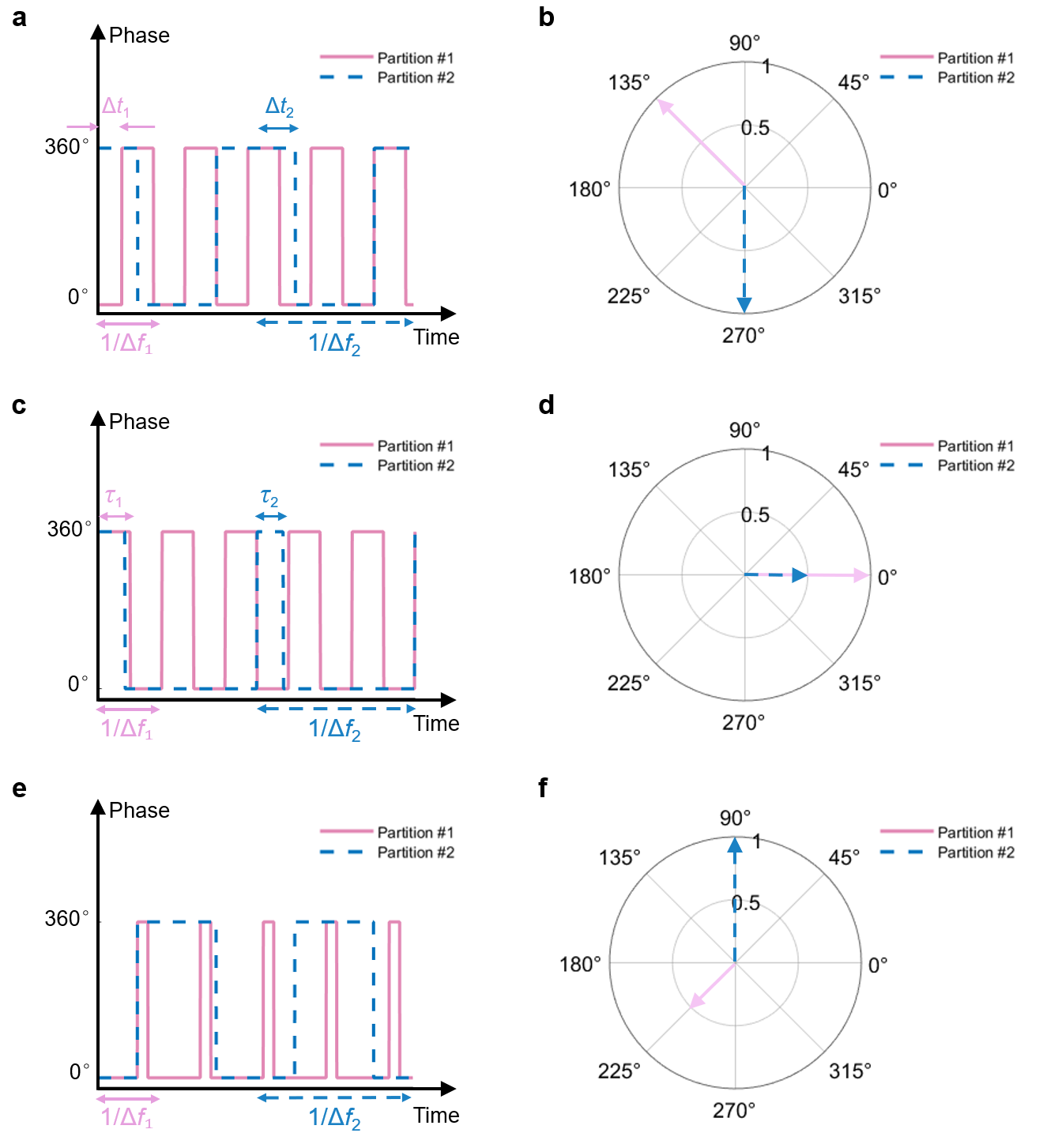


**Supplementary Figure S10 | Time varying reflection coefficients and the corresponding phasor diagrams for the frequency components *f*_1_ (pink solid lines) and *f*_2_ (blue dashed lines). a** Equal duty ratio with different time delays. **b** *A*_1_ = *A*_2_ = 1, *φ*_1_*=*135° and *φ*_2_*=*270°. **c** Equal time delay with different duty ratios. **d** *A*_1_ = 1, *A*_2_ = 0.5, and *φ*_1_*=φ*_2_*=*0°. **e** Different time delays with different duty ratios. **f** *A*_1_ = 0.5, *A*_2_ = 1, *φ*_1_*=*225°, and *φ*_2_*=*90°.


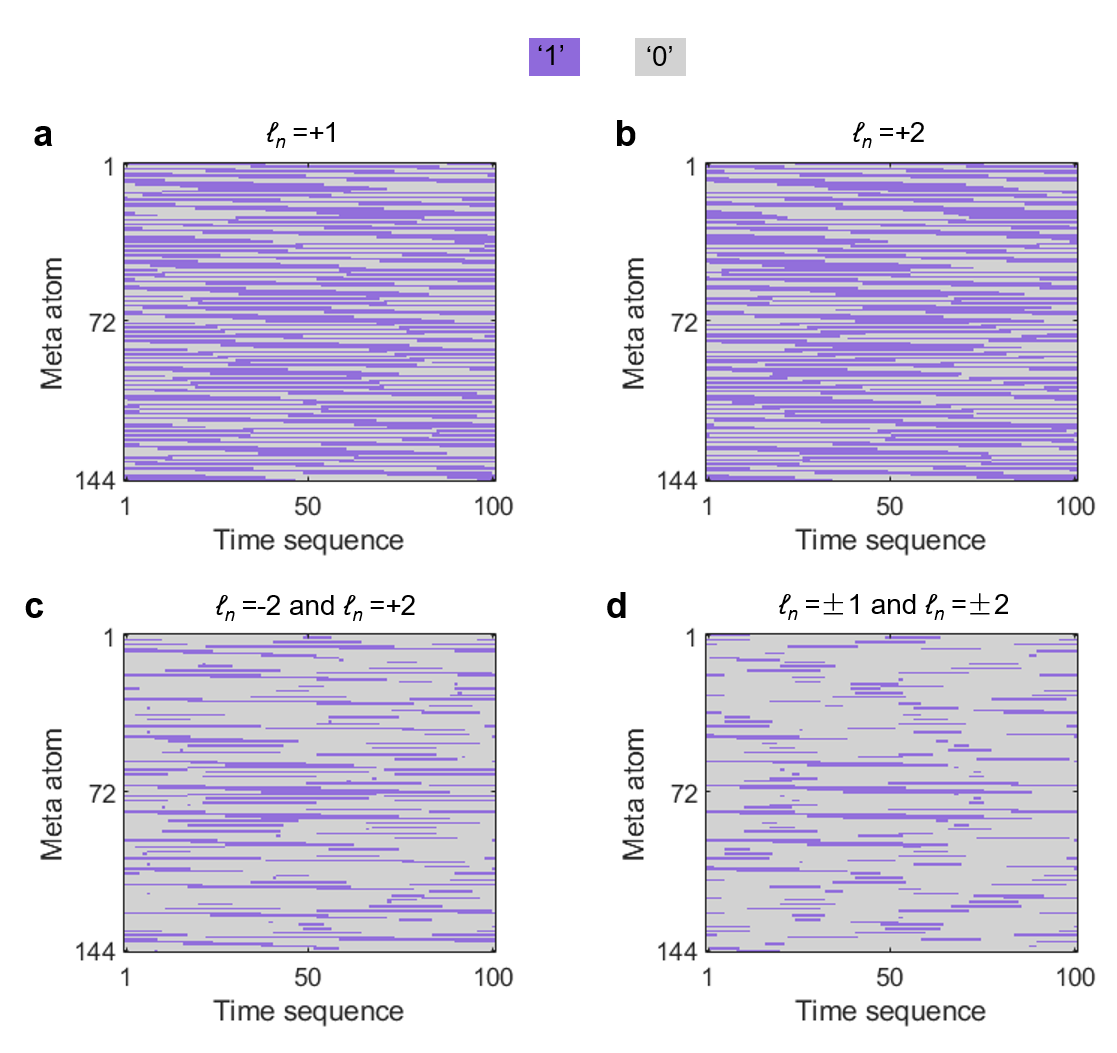


**Supplementary Figure S11 | STM matrix for vortex beams generation. a** OAM mode *ℓ_n_*=+1. **b** OAM mode *ℓ_n_*=+2. **c** OAM mode *ℓ_n_*=±2*.* **d** OAM mode *ℓ_n_*=±1, ±2.


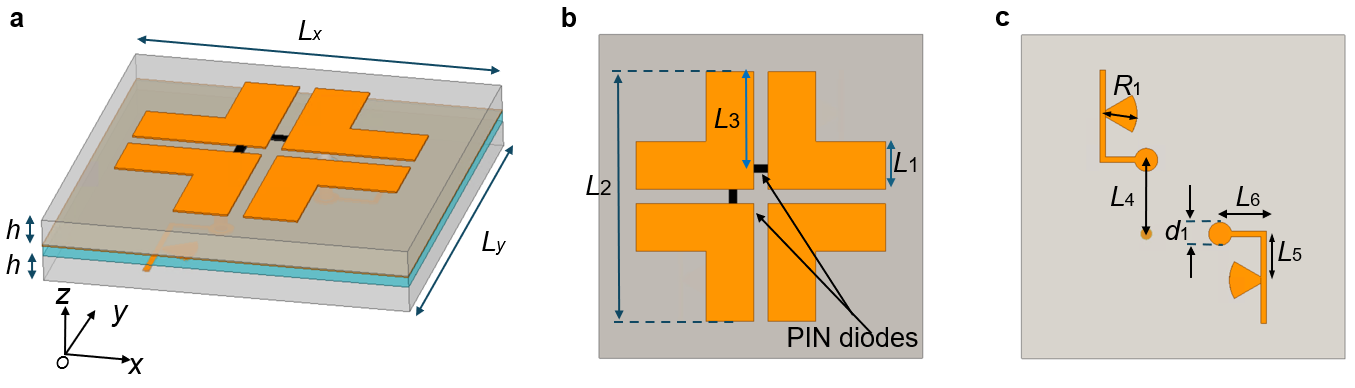


**Supplementary Figure S12 | Configuration of the proposed dual-LP meta-atom. a** Perspective view. **b** Top view. **c** Bottom view.

**References**

Table S4| Dimensions of the dual-LP meta-atom loaded with PIN diodes

| Parameters | *L_x_* | *L_y_* | *L*_1_ | *L*_2_ | *L*_3_ | *L*_4_ | *L*_5_ | *L*_6_ | *d*_1_ |
| --- | --- | --- | --- | --- | --- | --- | --- | --- | --- |
| Value (mm) | 7 | 7 | 1.04 | 5.4 | 2.1 | 1.6 | 1 | 1 | 0.5 |
| Parameters | *R*_1_ | *h* |  |  |  |  |  |  |  |
| Value (mm) | 0.8 | 0.508 |  |  |  |  |  |  |  |

**Supplementary Figure S13 |** Measured SNR versus transmission distance under the condition of OAM mode *ℓ_n_* =+1, *x*-polarization and 26.8001GHz.

1. Sun, C., Yang, S., Chen, Y., Guo, J. & Qu, S. Realization of multiple orbital angular momentum modes simultaneously through four-dimensional antenna arrays. *Scientific Reports* **8**, 149 (2018).

2. Wang, J. et al. Terabit free-space data transmission employing orbital angular momentum multiplexing. *Nature Photonics* **6**, 488-496 (2012).

3. Zhang, W. et al. Mode Division Multiplexing Communication Using Microwave Orbital Angular Momentum: An Experimental Study. *IEEE Transactions on Wireless Communications* **16**, 1308-1318 (2017).

4. Yang, H. Q. et al. Adaptively programmable metasurface for intelligent wireless communications in complex environments. *Nature Communications* **16**, 6070 (2025).

5. Zhou, Q.Y. et al. Two-dimensional direction-of-arrival estimation based on time-domain-coding digital metasurface. *Applied Physics Letters* **121** (2022).

6. Venkatesh, S., Lu, X., Saeidi, H. & Sengupta, K. A high-speed programmable and scalable terahertz holographic metasurface based on tiled CMOS chips. *Nature Electronics* **3**, 785-793 (2020).

7. Kim, M. et al. Monolayer molybdenum disulfide switches for 6G communication systems. *Nature Electronics* **5**, 367-373 (2022).

8. Zhang, Y. et al. Electrically reconfigurable non-volatile metasurface using low-loss optical phase-change material. *Nature Nanotechnology* **16**, 661-666 (2021).
